# Facilitating factors and barriers to kangaroo mother care utilisation in low- and middle-income countries: A scoping review

**DOI:** 10.4102/phcfm.v13i1.2856

**Published:** 2021-08-23

**Authors:** Christina T. Mathias, Solange Mianda, Julius N. Ohdihambo, Mbuzeleni Hlongwa, Alice Singo-Chipofya, Themba G. Ginindza

**Affiliations:** 1 Discipline of Public Health, School of Nursing and Public Health, University of KwaZulu-Natal, Durban, South Africa; 2 Department of Public Health, School of Public Health, University of the Western Cape, Cape Town, South Africa; 3 Department of Engineering and Public Health, Faculty of Health & Biomedical Sciences, Melbourne Institute of Technology University, Bundoora, Australia

**Keywords:** barriers, facilitating factors, kangaroo mother care, utilisation, low-birth-weight infants, parents

## Abstract

**Background:**

Kangaroo mother care (KMC) has been widely adopted in low-and middle-income countries (LMICs) to minimise low birthweight infants’ (LBWIs) adverse outcomes. However, the burden of neonatal and child mortality remains disproportionately high in LMICs.

**Aim:**

Thus, this scoping review sought to map evidence on the barriers, challenges and facilitators of KMC utilisation by parents of LBWIs (parent of low birthweight infant [PLBWI]) in LMICs.

**Methods:**

We searched for studies conducted in LMICs and published in English between January 1990 and August 2020 from SciELO, Google Scholar, JSTOR, LILACS, Academic search complete, PubMed, CINAHL with full text, and Medline databases. We adopted Arksey and O’Malley’s framework for conducting scoping reviews. Potential studies were exported to Endnote X7 reference management software for abstract and full article screening. Two independent reviewers did a parallel abstract and full article screening using a standardised form. The results were analysed using thematic content analysis.

**Results:**

We generated 22 040 studies and after duplicate removal, 42 studies were eligible for full-text screening and 22 studies, most form sub-Saharan Africa, were included in the content analysis. Eight themes emerged from the analysis: access, buy-in, co-ordination and collaboration, medical issues, motivation, social support-gender obligation and empowerment, time and timing and traditional/cultural norms.

**Conclusion:**

Identifying factors affecting KMC may optimise KMC utilisation. Additional studies aiming at identifying influencing factors that affect KMC utilisation amongst PLBWIs’ in LMICs need to be conducted to provide evidence-based strategies to enhance practice, inform policy and decision-makers in KMC utilisation amongst the PLBWIs in LMICs and beyond.

## Background

Kangaroo mother care (KMC) is a cost-effective, evidence-based intervention useful for preventing or reducing complications and reducing neonatal mortality in the preterm birth and low birthweight infants’ (LBWI), weighing 2400 g and below, regardless of their gestation age at birth.^1,2,3,4,5,6^ Low birthweight (LBW) is associated with intrauterine growth interruption related to preterm birth of a baby, usually with birthweight of 2500 g and below.^7,8^ Low birthweight can also manifest in the mature birth of a growth restrained foetus, hence, the words LBW and preterm birth can be used interchangeably.^9^ In this review, LBW is used to refer to babies with birthweight of less than 2500 g, parent of low birthweight infant (PLBWI) is referred to a caregiver who is either a father, mother or family member, to the LBWI. Classification and definition of low- and middle-income countries (LMICs) are by World Bank; low-income status based on gross national income (GNI) per capita of $1045.00 or less.

### Impact of kangaroo mother care on neonatal mortality

Globally, 90% of the annual registered LBWIs are born in LMICs of which 50% – 80% of these births contribute to the global neonatal mortality.^10,11,12^ Furthermore, preventable LBWIs deaths have been the leading cause of neonatal mortality contributing to more than 60% of neonatal deaths.^10,13^ To address this burden, KMC was introduced and globally adopted to manage LBWIs’ complications, which has proven to be effective.^14^ In LMICs KMC intervention is used in place of incubators because of the limited resources that do not allow incubator care facilities to be availed in primary healthcare facilities.^4,10,15,16,17,18,19^ Since its inception, KMC intervention has reduced 50% of the LBWIs’ deaths in LMICs because of its numerous advantages on LBWIs. These are warmth, promoting exclusive breastfeeding and growth.^10,15,20,21,22^ Although LMICs have implemented KMC widely over the past decades^4,15,16,18,19^ a limited number of reviews have been conducted to assess barriers and enablers to KMC. Furthermore, few studies have highlighted caregivers, health providers and health systems perspectives and/or recommendations that might be critical for enhancing KMC utilisation amongst PLBWIs.^23,24,25,26^ Given the pronounced burden of neonatal mortality attributed to LBWIs’ neonatal and child mortality in LMICs, utilising the much-needed KMC intervention to alleviate this burden remains an urgent challenge.^3,13,15,18,21^ To address this burden this review sought to identify the facilitating factors and barriers to the utilisation of KMC focusing on the PLBWIs, who are the key users to KMC. Utilisation of an intervention by the intended population is one of the monitoring and evaluation measures of an intervention’s success, which uncovers factors contributing to success and/or barrier to the utilisation of an intervention.^27,28^ Here, we identified evidence-based approaches to steer policy development, facilitated KMC uptake by the PLBWIs,

## Methods

### Study design

We reviewed studies to identify the barriers, challenges and facilitating factors of KMC utilisation by PLBWIs from LMICs. In particular, we mapped evidence-based facilitating factors and barriers to KMC utilisation by the PLBWIs and identified KMC utilisation knowledge gaps.^29^ We adopted the PRISMA-ScR Checklist to systematically assess the scope of literature^30^ (Appendix 1). We also adopted Arksey and O’Malley methodological framework, which entailed the following stages:^31^

- Identifying the research question
- Identifying relevant studies
- Study selection
- Charting the data
- Collating, summarising and reporting

### The research questions

The protocol guiding this scoping review has previously been published.^32^ The main review question sought to identify factors that facilitate and hinder the utilisation of KMC by PLBWIs in LMICs. The review’s specific questions were:

- What are the factors influencing KMC utilisation amongst parents of PLBWIs in LMICs?
- What are the barriers for KMC utilisation amongst parents of PLBWIs in LMICs?
- What are the experiences of mothers of PLBWIs in utilisation of KMC in LMICs?

### Eligibility of the research question

The Sample, Phenomenon of Interest, Design, Evaluation and Research type (SPIDER) framework was used to determine the eligibility of the studies.^33^ (Table 1)

**TABLE 1:** Framework determining the eligibility of the research question.

Criteria	Determinant
Sample	Parents/guardian of LBWIs utilising KMC
Phenomenon of interest	Kangaroo mother care
Design	Randomised control clinical trials; non-randomised experiments; survey; cross-sectional, case-control and cohort studies
Evaluation	Barriers, challenges, bottlenecks, enablers, experiences and facilitating factors to KMC utilisation
Research type	The qualitative, quantitative and mixed-method

*Source*: Adapted from Cooke A, Smith D, Booth A. Beyond PICO. Qual Health Res. 2012;22(10):1435–1443. https://doi.org/10.1177/1049732312452938

LBWIs, low birthweight infants; KMC, Kangaroo mother care.

### Identified studies

The review included qualitative, quantitative and mixed-method primary research studies published in peer-reviewed journals and grey literature that addressed the research question. The review included the following study designs: cross-section studies, randomised controlled trials, formative, phenomenological and survey-descriptive studies. The electronic databases used to search for the relevant studies were: Academic search complete, Cumulative Index of Nursing and Allied Health Literature (CINAHL) with full text, PubMed, Education source, Health Source: Nursing/Academic Edition, Medline with full text and Medline. All these electronic databases accessed individually via the Elton B. Stephens Company (EBSCOhost) search engine. We also searched studies from the Scientific Electronic Library Online (SciELO) and the Latin-American and Caribbean System on Health Sciences Information (LILACS) databases. Google Scholar search engine, Journal Storage (JSTOR) search engine, ‘the cited by’ and reference lists were used to search for the relevant literature. Studies wrote in English and automatically translated into English were reviewed.

Low- and middle-income countries have been implementing KMC since its introduction in 1978 by Ray; however, we only included studies published between January 1990 and August 2020. The United Nations marked 1990 as a baseline year for the Millennium Development Goals^34^ and as such, we elected to use this year as our baseline for the review. The search terms of this scoping review originated from indexed subject headings, keywords of relevant studies, terms from this scoping review that recurred repetitively and the Medical Subject Headings (MeSH) terms. The search terms included (‘Kangaroo mother care’ OR ‘kangaroo care’ OR ‘skin to skin’ OR ‘kangaroo-mother care method’ OR ‘skin to skin contact’) AND (‘parents’ OR ‘mother’ OR ‘father’ OR ‘family caregivers’) AND (‘low birthweight infants’ OR ‘preterm infants’ OR ‘premature infants’ OR ‘very low birthweight infants’) AND (‘utilisation OR “uptake” OR compliance’) AND (‘facilitators’ OR ‘enablers’ OR ‘motivators’ OR ‘experience’ OR ‘perception’ OR ‘attitudes’). The identified studies were screened using the eligibility criteria. Table 2 indicates the pilot electronic database search.

**TABLE 2:** Pilot electronic database search strategy.

Search terms	Database	Search results
((((((kangaroo mother care) OR (skin to skin contact)) AND (mother)) AND (low birthweight infant)) OR (preterm infants)) AND (enablers)) AND (utilisation) Filters: from 01 January 1990 to 31 August 2020	PubMed	225


### Study selection criteria

Here is the review’s eligibility criteria; inclusion and exclusion criteria.

#### Inclusion criteria

The studies included in the review were:

- Studies published in English and in other languages with the English version.
- Studies on factors that influence the utilisation of KMC by PLBWIs in LMICs.
- Studies focusing on experience, views or perception of PLBWIs on the utilisation of KMC in LMICs.
- Studies with the given criteria and published between January 1990 and August 2020.

#### Exclusion criteria

The studies excluded in the review were:

- Studies published in languages other than English without an English version
- Studies published before January 1990 or after August 2020 regardless of being eligible
- Studies conducted in high-income countries regardless of being eligible
- Studies with a phenomenon of incubator care conducted in LMICs or high-income countries
- Systematic review, as they summarise included primary studies.

### Study selection procedure

This scoping review involved two phases, as follows:

**Phase 1:** One reviewer performed title screening from the proposed databases, by examining the relevance of the study titles to the research purpose. The identified studies imported to Endnote X7 reference management software and duplicates removed. The Endnote X7 library was shared with the two reviewers, who in parallel independently screened the studies’ abstracts according to the eligibility criteria. The full text of the eligible studies retrieved and imported to EndNote X7 library. The reviewers consulted the University of KwaZulu-Natal librarian to assist with the missing five full texts, which were not available.

**Phase 2**: Two reviewers independently performed a parallel full article screening, following the eligibility criteria and excluded the studies with reasons. There was a discrepancy with the eligibility of one article, which was resolved by the third reviewer. The study was not included as it was conducted in a high-income country. Data extraction was performed on the eligible studies that were identified during the full article screening. Two reviewers extracted data in parallel and independently, using the data charting form as presented in Box 1. Reviewers shared notes during abstract and full article screenings. Throughout the selection of eligible studies, the reviewers kept account of the number of the studies imported to the Endnote X7, the number of duplicates removed and the number of eligible studies for the abstract screening. During the abstract screening, the reviewers took note of the number of the excluded studies, indicating the reason for exclusion. The number of studies eligible for full article screening and the number of studies excluded during full article screening, indicating reasons, were recorded. Figure 1 shows a summary of the study selection process.

Box 1 Data charting form.

Author and date

**Title of the study**

Aim of the study/research question

**Population**

- Sample size
- Characteristics of participants
  ▪ Percentage (%) and number of males
  ▪ Percentage (%) and number of women
  ▪ Age/average

**Intervention**

**Study design**

**Recruitment setting**

**Sampling strategy**

**Data collection (methodology)**

**Data analysis**

**Outcome of the study/results**

**Conclusion of the study**

**Significant findings**

**Comments**

*Source*: Adapted from Mathias CT, Mianda S, Ginindza TG. Evidence of the factors that influence the utilisation of kangaroo mother care by parents with low-birth-weight infants in low- and middle-income countries (LMICs): A scoping review protocol. Syst Rev. 2018;7:55. https://doi.org/10.1186/s13643-018-0714-9

**FIGURE 1:**
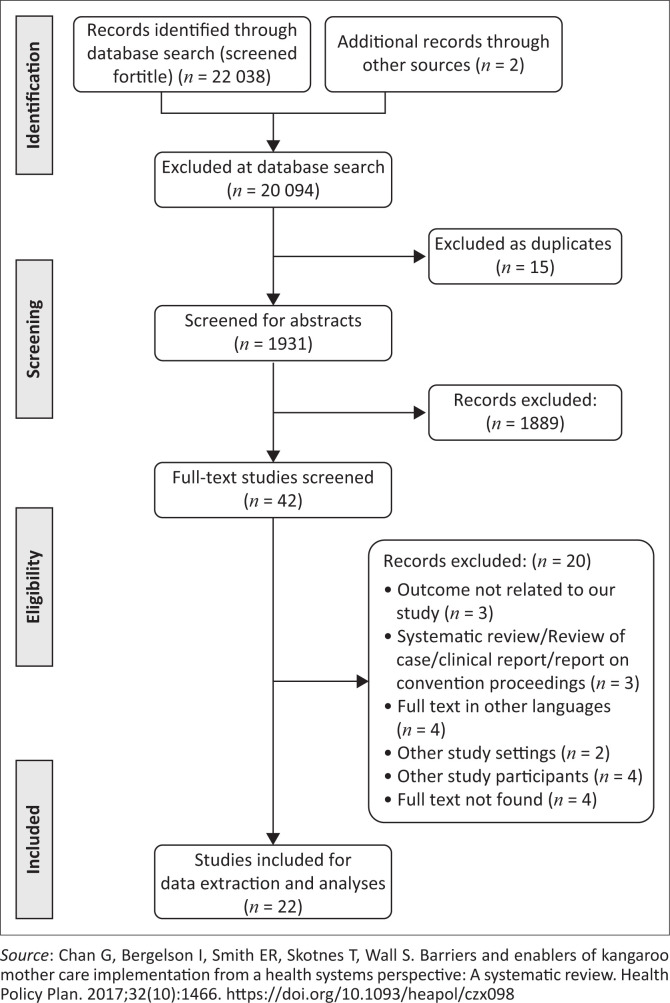
Prevention and Recovery Information System for Monitoring and Analysis (PRISMA) flow diagram.

### Data charting

The standardised data extraction was guided by the data charting form (Box 1), which was electronically piloted on a google form and regularly updated, to address the research question. The challenges of KMC utilisation, barriers to KMC utilisation and facilitating factors of KMC utilisation were the study outcomes and experiences that guided data extraction. The coding of arising themes and narrative analysis of the extracted data was performed by the thematic content analysis.

### Collate, summarise and results reporting

The stages included coding text, developing descriptive themes and generating analytical themes.^35^ These three stages were interrelated in such a way that the free coding of the primary study findings facilitated the organisation of the codes into descriptive themes that enhanced the development of analytical themes.

#### Stages 1 and 2: Coding text and descriptive themes development

Two reviewers independently and in parallel did line-by-line coding of the primary study findings, regarding the context and meaning. However, the coding of the study’s findings did not strictly depend on the research question, as few studies were addressing the review question directly.^35^ The reviewers then categorised the initial codes into major groups, depending on their similarities and differences, then, new codes were assigned to the grouped codes to give a descriptive meaning to the groups, hence, the development of descriptive themes. One reviewer wrote a draft summary of the descriptive themes, which was reviewed by the rest of the reviewers and agreed on the final version of the descriptive themes.

#### Stage 3: Generating analytical themes

The reviewers deduced the barriers, challenges and facilitating factors of KMC utilisation by PLBWIs from the descriptive themes, which was conducted independently and in parallel. At this stage, the reviewers individually analysed the descriptive themes and examining the relationship between themes to the review question. The reviewers examined the meaning of the study’s findings to the review question. The implications of the findings were considered for intervention development. Through the narrative analysis process, the analytical themes were developed and interventions proposed. The reviewers had a group discussion on the review question and the implications of the descriptive themes, where the analytical themes and implications for intervention development emerged. The process was repeated until we no longer had emerging analytical themes and implications for intervention development. The reviewers summarised stage three by agreeing and approving the identified analytical themes and implications for intervention development. The implications formed the recommendations of the review.

### Data analysis

The included studies were appraised based on the discussions by the authors. Areas of disagreement were mutually resolved through a discussion. A thematic content analysis was conducted using the following themes: facilitating factors and barriers to KMC utilisation. The results are presented using a narrative approach and the emerging themes were reported.

## Results

### Study selection process

The electronic search strategy identified 22 040 studies, which were screened for titles (Figure 1). The 20 094 studies were not selected during the database search stage because they did not meet the inclusion criteria. Fifteen duplicates were removed, leaving 1931 studies, which were screened for abstracts. A total of 1889 studies were removed at the abstract screening stage because they met the exclusion criteria. The researchers further screened 44 full-text studies of which 20 studies were excluded because three studies’ outcome were not related to our study, three were systematic review, review of case, clinical report and report on convention proceedings, four had full text in other languages, two had other study settings, four had other study participants and in four studies full text was not found. Therefore, 22 studies met our inclusion criteria and were included in content analyses.

### Included studies characteristics

The 22 included studies reported on factors influencing and barriers to KMC utilisation amongst PLBWIs mostly published between 2012 and August 2020. The included studies comprised four cross-section studies^36,37,38,39^ three randomised control trial studies,^40,41,42^ one explorative study^43^ seven descriptive studies,^44,45,46,47,48,49,50^ one longitudinal study,^49^ one qualitative study,^51^ two dissertations^39,52^ and two formative study.^47,53^ Almost 10 studies were based on face-to-face interviews,^36,37,38,43,44,45,46,47,48,50^ of which seven were qualitative study interviews,^36,37,43,47,48,50,53^ three were mixed-method studies^38,44,46^ and nine were quantitative studies. Out of the 22 studies, 19 were conducted in the facility^36,38,39,40,41,42,43,44,45,46,48,49,50,51,52,54,55,56^ and three were community-based involving mothers, fathers, grandmothers and traditional birth attendants (TBA).^37,47,53^ A total of 30% of the studies had a sample size of > 13, 60% of the studies had a sample size range of 28–349. Of the 22 studies, one was conducted in Bangladesh, one in Brazil, two in Ethiopia, two in Ghana, three in India, one in Indonesia, five in Malawi, one in Mozambique, one in Nigeria, one in Pakistan and four studies in South Africa (Table 3).

**TABLE 3:** Description of characteristics of the included studies in assessing kangaroo mother care utilisation in low- and middle-income countries, 1990–2020.

Number	Author	Setting	Characteristics of the participants	Sample size	Female	Male	Study design/methodology	Significant findings related to our study
1	Arivabene et al. ^44^	Espirito Santo State, Brazil	Mothers of low weight preterm infants	13	13	0	Descriptive study; qualitative & quantitative	Family support in KMC practice is essential to the success of KMC utilisation
2	Opara and Okorie ^45^	University of Port Harcourt Teaching Hospital, Nigeria	Mothers who had practised KMC and whose LBWIs had been discharged from the Special Care Baby Unit	42	42	0	Descriptive study; quantitative	Ongoing KMC health talks facilitate KMC utilisation. Parents who know KMC comfortably practice KMC than mothers who have limited or no knowledge on KMC
3	Roba et al. ^38^	Dilchora and Hiwot Fana, Ethiopia	Postnatal mothers of preterm and low birthweight babies	349	349	0	Descriptive cross-sectional study; Mixed method (face-to-face interview and questionnaire)	Health education on KMC at antenatal clinic sessions may enhance complete acceptance after delivering a LBWI
4	Chisenga et al. ^46^	Lilongwe and Zomba Hospitals, Malawi	All mothers who had their preterm/LBW infants in the KMC unit at Bwaila Hospital in Lilongwe and Zomba Central Hospital in Zomba and those that had come for follow-up 2 weeks after hospital discharge before this study started.	113	113	0	Descriptive study; quantitative and open interviews	Lack of KMC knowledge amongst mothers before hospitalisation because of lack of community sensitisation hinders KMC acceptability after LBWI’s birth
5	Hunter et al. ^36^	Tungipara Subdistrict, Gopalganj District, Bangladesh	Pregnant women and mothers, husbands, maternal and paternal grandmothers, traditional birth attendants, village doctors, traditional healers, pharmacy men, religious leaders, community leaders	40	27	13	Cross-section study; Qualitative in-depth interviews (IDIs) and focus group discussions (FGDs)	Kangaroo mother care community sensitisation and promoting KMC promotional messages through the media and trained healthcare providers may help adoption, acceptability and accessibility of KMC by the influential community leaders and the mothers
6	Reddy and Mclnerney ^48^	KwaZulu-Natal, South Africa	Mothers who were practising KMC in the postnatal ward or mothers who were discharged and were still practising KMC	10	10	0	Descriptive study; qualitative	Support from nurses, fellow mothers and family enhances KMC acceptability and utilisation
7	Nguah et al. ^49^	Kumasi, Ghana	Mothers and their inpatient LBW neonates	202	202	0	Longitudinal study; quantitative	Follow-up of LBWI on KMC from admission, follow-up visits to discharge improves attitude and perception towards KMC practice by mothers
8	Bazzano et al. ^37^	Kintampo, Ghana	Mothers with LBWIs and traditional birth attendants	29	29	0	Cross-sectional study; in-depth interviews and focused group discussions	Kangaroo mother care practice is demonstrated as a new practice. For easy adoption of the new practice, KMC awareness and demonstration using dolls and photographs of the local women practising KMC would help in easy acceptability, acceptability and utilisation of KMC.
9	Angela Leonard and Mayers ^43^	Cape Town, South Africa	Parents who were actively involved in providing KMC to their preterm infants	6	4	2	Phenomenological study; qualitative, explorative	Fears, emotions and hopelessness undergone by mothers of LBWIs can be overcome by family, spouse and nurse’s support and encouragement.
10	Mazumder et al. ^47^	Faridabad and Palwal, in the state of Haryana, India	Mothers, grandmothers and fathers	36	28	8	Formative study; descriptive-in-depth interviews and focused group discussions	Family and community influencers’ support coupled with conducive and supportive environment enhance KMC utilisation by mothers
11	Ramanathan et al. ^40^	India	Mothers with LBWIs	28	28	0	Randomised control trial study; questionnaire-Likert scale	Kangaroo mother care practice is acceptable; its feasibility is granted in the hospital setting for hospital deliveries unlike the home deliveries
12	Maja and Kerstin ^50^	Maputo, Mozambique	Mothers with LBWIs	41	41	0	Descriptive study; face-to-face interview	Lack of health education, prior KMC awareness, inadequate skills by the nurses hinder informed decision making on KMC acceptability and utilisation
13	Mathias et al. ^54^	Mangochi, Malawi	Mothers with LBWIs	12	12	0	Descriptive study; focused group discussions	Inclusion of KMC messages in antenatal care guidelines, community awareness and sensitisation are key factors in enhancing KMC accessibility and utilisation by the targeted population
14	Yusuf et al. ^55^	Yirgalem, Ethiopia	Mothers with LBWIs	215	215	0	Cross-sectional study; qualitative	Ongoing KMC health education is crucial in the continuation of KMC practice at the community level
15	Kurniawati et al. ^39^	Jakarta, Bogor, Tangerang, and Bekasi, India	Mothers with LBWIs	24	24	0	Randomised control trial; quantitative	Peer support enhances KMC utilisation in both facility and community-based KMC.
16	Chavula et al. ^42^	Machinga, Thyolo, Blantyre, Malawi	Mothers with LBWIs	280	215	0	Randomised control trial; quantitative	Customised wrap supports KMC practice and it enhances confidentiality in PLBWIs
17	Jamali et al. ^51^	Sandh, Pakistan	Mothers of LBWIs and others	26	-	-	Qualitative study-IDIs and FGDs	Availability of resource and quality care service enhance KMC utilisation
18	Dawar et al. ^56^	Delhi, India	Mothers with LBWIs	60	60	0	Exploratory-observational study; mixed method	Ongoing KMC education and support may enhance KMC utilisation
19	Lydon et al. ^53^	Southern Malawi	Pregnant women, community members and women who had practiced KMC	152	-	-	Formative study; qualitative (FGDs and IDIs)	Targeted KMC education to pregnant and risk mother who are at risk of delivering LBWIs Strengthened partnership of community key influential people in KMC
20	Mathias ^52^	Southern Malawi	Mothers with LBWIs	50	50	0	Descriptive study; Quantitative (dissertation)	Kangaroo mother care’s support and knowledge enhanced KMC compliance
21	Solomons and Rosant ^39^	Cape Town, South Africa	Mothers with LBWIs and antenatal nurses	43	43	0	Descriptive cross-sectional study; quantitative	Targeted PLBWIs and pregnant women with KMC messages through health talks, KMC demonstration and distribution of flyers.
22	Solomons and Rosant ^39^	Cape Town, South Africa	Mothers with LBWIs and antenatal nurses	43	43	0	Descriptive cross-sectional study; quantitative (dissertation)	Kangaroo mother care’s messaging should be PLBWIs and pregnant women centred, disseminated through health talks, KMC demonstration and distribution of flyers.

*Source*: Torres NF, Chibi B, Kuupiel D, Solomon VP, Mashamba-Thompson TP, Middleton LE. The use of non-prescribed antibiotics; prevalence estimates in low-and-middle-income countries. A systematic review and meta-analysis. Arch Public Health. 2021;79(2):1–15.

PLBWIs, parent of low birthweight infant; KMC, Kangaroo mother care; LMICs, low- and middle-income countries; LBWIs, low birthweight infants; FGD, focus group discussions; IDI, in-depth interviews.

### Study’s findings

We identified eight themes that described facilitating factors and barriers to KMC utilisation by PLBWIs, (Table 4). The themes were *access* (availability of KMC providers, resources, place of delivery, maternal love, privacy, preference of KMC over incubator care, the season of the year and nurses’ empathy), *buy-in* (KMC knowledge, perceived and experienced KMC benefits, KMC awareness, attitude towards KMC and type of wrap), *co-ordination and collaboration* (infants’ health updates, educate key players and good rapport), *medical issues* (safety, infection, deformity and maternal health), *motivation* (use of expert clients, return demonstrations, KMC posters, recreational activities and observed KMC benefits), *social support, gender obligation and empowerment* (KMC support, encouragement, aid in KMC practice, male involvement, woman decision-making), *time and timing* (KMC initiation, limited visiting hours, long hospitalisation stay, KMC consumes time, KMC waste of time and timing of acquisition of KMC knowledge) and *traditional/cultural norms* (beliefs).

**TABLE 4:** Matrix for focus group discussions/in-depth interviews for facilitating factors and barriers to kangaroo mother care utilisation by parents of low birthweight infants in low- and middle-income countries, 1990–2020.

Theme	Facilitators	Barriers
**Access to KMC**		
Antenatal care	Health facility: Acquired KMC education	Home: Missed KMC education
Attitudes	Caregivers’ preference of KMC over incubator care Provider (nurse) empathy-promoted KMC health education	KMC providers unwillingness to support KMC
Maternal self-efficacy	Wanting to see the infant survive A sense of bonding between the mother and infant Mothers’ love/affection towards the LBWI gives zeal to practice Confidence in KMC practice Willingness to practice KMC Accepting KMC as a good strategy	Low self-esteem Lack of confidence in KMC practice PLBWI felt less of a woman Post-delivery weakness and pains
Place of delivery	Health facility: availability of KMC providers	Home: Difficult to identify LBWI, led to late/delayed initiation of KMC or KMC uptake
Privacy	-	Hospital: Spectators when males practice KMC Home: Limited space and extended family at home
Season of the year	Maximised KMC practice in winter	-
Resources	Health facility: availability of chairs, lighting and ventilation	Home: Lack of comfortable chair, ventilation and lighting
Type of wrapper	Customised wrap	-
Quality of care	-	Compromised Quality care: Documentation, monitoring and follow-up
**Buy-in**		
KMC benefits	Perceived/observed and experienced KMC benefits Satisfied with KMC benefits	No observed/experienced KMC benefits Brought fear and anxiety in mothers
KMC knowledge/awareness	Mothers: KMC protocol, support and features of LBWI Community: Spouse, family and community	Mothers: LBWI features, KMC protocol and safety of the LBWI –Community: Influential people and the community
**Coordination and collaboration**		
Nurse-mother rapport	Enhanced combined efforts in KMC Promoted infant’s health updates Enhanced empathy to KMC mothers or infants by nurses	Refrained infants’ health updates Demotivated mothers to practice KMC
Capacitate key players in KMC	Traditional birth attendants Grandmothers	-
**Medical issues**		
KMC Safety	KMC as an intervention KMC practiced by inactive mothers	Umbilical cord stump injury and bleeding LBWI may slip off the chest Obstructs LBWI airway Mothers sleep on LBWI Exposes LBWI to harsh weather Skin rash and umbilical cord infection Causes neck deformity
Maternal health	-	Post-delivery weakness and pains
**Motivation**		
Maternal discomfort with KMC	Being the first-time mother Feeling chest pains and backache with KMC practice	-
KMC outcomes	Observed, witnessed or experienced KMC benefits motivated PLBWI	-
Recreational activities	Watching TV removes boredom on PLBWIs	Brings a feeling of confinement, which brought boredom and loneliness
Return demonstrations	Using dolls and KMC pictorial presentations	-
Use of expert clients	Shared positive KMC experiences	-
KMC posters/pictures	Visual posters: motivated mothers to practice KMC	-
Maternal social life	-	Increased house-workload Brought confinement Disturbed social/employment life
KMC follow-up	Hospital: Guidance on KMC interventions at facility-based KMC	Community interventions: lack of guidance on KMC practice at home
**Social support, gender obligation and empowerment**		
KMC encouragement and support	Spouse, relatives, community or fellow PLBWI with house chores and/or KMC practice KMC support groups motivated or encouraged PLBWIs to practice KMC Prior identification of support system facilitated KMC support Encouragement of KMC health workers and fellow PLBWIs on KMC continuity Hospital: KMC health providers support with KMC initiation and health education	Non-supportive spouse/relatives with KMC practice Difficult to do KMC with twins with no family support Home: Non-continuation of KMC health education at community-based KMC
Male involvement	Brought infant–father bonding and father’s confidence and will to practice KMC	Males critiqued by mother on KMC practice Mothers’ not comfortable with infants under male care Lack of male inclusion in KMC unit setup Males denied spending time in KMC unit
Social or gender obligations		KMC conflicts with social or gender obligations prevents mothers from practising KMC consistently
Women empowerment	Hastened KMC utilisation	Lack of women empowerment: Delays decision in KMC initiation
**Time and timing**		
KMC initiation	Timely KMC initiation: KMC providers’ support with early KMC initiation	Late KMC initiation: Medical stabilisation of LBWIs Delayed KMC support rendered to PLBWI Late decision making by PLBWIs to initiate KMC Waiting for the umbilical cord to fall
KMC unit visiting hours	-	Limitation: Family members not to stay for long in the KMC unit
KMC practice duration	-	Hospitalisation: KMC infants take a long time before they are discharged KMC consumes time: conflicts with gender responsibilities KMC is waste of time: KMC is for the whites
Timing of acquisition of KMC knowledge	-	Lack of KMC education at antenatal and KMC unit – missed opportunity for KMC sensitisation to the targeted population
Traditional/cultural norms		
Customary attire	Front open ascribed traditional: similar to KMC recommended attire	Front closed ascribed traditional: deemed conflicting with KMC attire
Maternal cultural practice	Confinement after delivery: promoted KMC practice and mother–infant bonding	-
KMC perception	-	KMC considered a taboo: Unwilling to incorporate KMC as a new practice KMC defiled traditionally ascribed normal way of carrying an infant Ridiculed by the community for giving birth the LBWI PLBWI felt less of a woman

PLBWIs, parent of low birthweight infant; KMC, Kangaroo Mother Care; LMICs, low- and middle- income countries; LBWIs, low birthweight infants.

#### Access to kangaroo mother care utilisation

This review found that availability of skilled health workers,^45^ maternal love, infant survival, health-seeking behaviour,^36,44,46,54^ preference of KMC over incubator care and referral of LBWIs to a tertiary health facility for further management^40,54^ facilitated access to KMC. This review further identified barriers to KMC utilisation; lack of privacy with KMC practice at home, especially in extended families where privacy is limited,^47^ and lack of privacy in the hospital by the male support system practising KMC.^40,56^ Males felt uncomfortable practising KMC in the hospital because of spectators as there were several beds in one room for KMC mothers.^43^ Availability of resources in the hospital promoted KMC utilisation unlike at home where, for example, chairs with backrest, ventilation and lighting to use when caring for the LBWI are limited.^37,47,51^ In the community set up, the mothers with LBWIs are ridiculed by mothers with a full-term infant who breed fear and discomfort to those practicing KMC in the community.^36,37,43,49^

Home delivery posed a challenge to identify LBWI as the infants are not weighed, the size of the infant was by comparison with the previous deliveries, which shows the missed opportunity to initiate and practice KMC at home.^36,40^ Low birthweight infant birth psychologically disturbed mothers, because of lack of KMC health education during antenatal clinics and lack of psychological and/or emotional preparedness of giving birth to the LBWI, which barred the mothers from KMC knowledge and KMC access.^39,43,53^ Dissemination of KMC information to mothers with LBWIs depended on the nurses’ willingness, kindness and empathy and these affected KMC utilisation.^50^ Season in which the mother delivered played a role in KMC practice, in winter mothers performed KMC than in summer.^36^ Mothers developed low self-esteem because of being wet with breastmilk, as KMC facilitated exclusive breastfeeding and milk leakage.^37^ Compromised quality care because of poor documentation, monitoring, follow-up and inadequate skills by KMC provider barred KMC utilisation by PLBWIs.^51,54^

#### Buy-in

Eleven of the included studies indicated that *KMC knowledge* affected acceptability and utilisation of KMC.^36,37,38,39,40,45,46,47,49,50,53^ Studies conducted in Ethiopia and Nigeria indicate that knowledge on KMC protocol, inclusion of support system and correct infant positioning promoted the utilisation of KMC in 30% of the mother who knew about KMC position and support. Lack of KMC knowledge in 70% of the mother in KMC position, duration and the type of clothes for an infant on KMC, barred KMC utilisation.^38,45^ An Indian study further explained that parents who knew how a LBWI looks like (tiny features) prompted acceptance and provided the infants with KMC and lack of knowledge on the LBWI’s features, in some mothers, deterred KMC initiation.^47^ Other studies conducted in Malawi, South Africa, Ethiopia, Mozambique and Ghana found that a lack of knowledge on KMC protocol by mothers and family members, safety of LBWI on KMC was a challenge in KMC utilisation.^37,38,39,50,53^ Lack of KMC knowledge by the community influential people; leaders or elders barred KMC recognition and acceptance as an intervention for LBWIs.^36,47,53^ Ten reviewed studies indicate that *KMC benefits* promoted KMC utilisation,^36,38,40,43,44,45,49^ of which only two studies found out that anticipated KMC outcomes prompted mothers to practice KMC.^38,44^ Five studies found that experienced positive results, infant’s weight gain, gave the zeal to practice KMC^40,43,45,47,48^ and three studies indicate that both perceived and observed KMC benefits promoted KMC utilisation.^36,46,49^ Satisfaction and feeling of accomplishment with KMC outcomes played a role in utilising KMC.^40,43,45,46^ However, not experiencing improvement on an infant on KMC and feeling afraid, anxious and confusion doubted the workability of KMC.^43,48^ *Kangaroo mother care awareness* by the spouse, family members and community promoted KMC acceptability and utilisation.^37,49^ However, studies conducted in Malawi, India, Mozambique found KMC is not known by the majority of the community, the lesser community got KMC information from non-profession persons, that was attributed by lack of community sensitisation and health education by service providers.^40,46,50,53^ *Positive attitude* towards KMC practice, willingness to practice, confidence in handling a LBWI and accepting KMC as a good strategy by mothers, promoted KMC utilisation,^36,38,39,40,49^ although, some study found that lack of mothers’ confidence and interest in practising KMC barred KMC practice.^38,40^ In Malawi, PLBWIs accepted the use of customised wrap because of its easiness to use, it promoted breastfeeding and KMC practise whilst sleeping as compared with a traditional wrap – *chitenje,*^42^ as such, use of customised CarePlus wrap promoted KMC utilisation than *chitenje.*

#### Coordination and collaboration

Mother–nurses relationship facilitated KMC practice, in which nurses’ empathy and love towards parents with LBWIs enhanced KMC practice.^43^ Educating the key players in the society; TBAs and grandmothers, enhanced acceptability and continuity of community support in KMC practice.^37,53^ Daily updating mothers on the condition of the infant by the nurses enhanced combined effort and facilitated KMC utilisation.^50^ However, mothers who were not involved in their infants’ healthcare decision-making and not updated on their infants’ health had no interest to perform KMC,^50^ hence they were barred from KMC utilisation.

#### Medical issues

Feeling comfortable and safe with an infant in KMC position promoted the utility of KMC.^40,45,46^ A study concurred that LBWI was safe on KMC position with the mothers who perform inactive chores, that is, knitting.^36^ However, some studies found PLBWI fear that KMC causes infant’s neck deformity, due to the neck positioning on KMC.^47^ Culturally, when the umbilical cord stump prolonged in contact with the mother’s skin, sweat causes umbilical cord stump injury, bleeding and skin rash and infection.^36,37,47^ Kangaroo mother care was considered unsafe by mothers as they feared LBWI can slip off the mother’s chest and KMC may obstruct the infant’s airway causing breathlessness.^36,37,43,48,49^ Others feared that a sleeping mother could roll on the infant. Kangaroo mother care was feared for exposing the infant to cold and heat when performing house chores, as such KMC was perceived as not a safe intervention for LBWIs. Some mothers indicated that postpartum pains and weakness hindered KMC utilisation.^47,52,55^

#### Motivation

Use of KMC expert clients enhanced KMC utilisation by mothers who were in a similar situation.^36^ In Malawi, Ghana and India, the use of KMC pictures or dolls, pictorial presentations and photographs/posters of mothers practising KMC and return demonstration of KMC dolls eased understanding of KMC practice and motivated mothers to practice KMC.^37,46,47^ Watching television motivated mothers to indulge in KMC practice although, some mothers felt lack of recreational activities and demotivated them to practice KMC.^46^ When mothers observed and witnessed KMC benefits from mothers practising KMC, it motivated the mothers to dedicate their time in KMC practice.^36^ Mothers’ love or affection towards the LBWI gave the zeal to practice KMC.^48^

The feeling of mother–infant contact brought comfort, hope and courage to continue with KMC.^40,43,48^ Nevertheless, some mothers felt discomfort with KMC position, especially first time and teenage mothers.^36,46^ The feeling of the infant not been comfortable in KMC position, the chest pains and backache experienced by the mother during KMC practice, demotivated mothers to continue with KMC practice.^36,37,47^ Some mothers viewed KMC to be increasing their household workload, disturbing their social life; interrupting employment and leisure, and the feeling of confinement brought boredom and loneliness,^43^ barring the uptake of KMC. Lack of home routine guidance on KMC practice as compared with facility KMC demotivated mother from practising KMC.^50^

#### Social support, gender obligations and empowerment

Family support with *KMC practice*, taking care of the other children, KMC *encouragement and supportive spouses* were identified as the facilitating factors to KMC utilisation by 11 of the reviewed studies.^36,39,43,44,45,47,48,49,50,52,53,56^ Nonetheless, lack of family support with KMC practice emerged as a barrier to KMC uptake in other studies^44,45,46,49^ and having twins with no support was a huge challenge to KMC utilisation.^47^ Community support with household chores, KMC encouragement, health workers’ support during KMC initiation, KMC support groups motivation, prior identification of KMC support system were identified as aides to KMC utilisation.^36,45,49^ Encouragement and support from fellow mothers practising KMC, nurses’ encouragement and support with on-going KMC health education facilitated KMC uptake.^37,40,41,43,44,46,48,50^ However, lack of continuous KMC health education was identified as a barrier to KMC utilisation.^45,46^

Male involvement in KMC brought confidence in the fathers who provided care to LBWI and had a will to bond with their LBWI, although mothers judged and criticised the fathers on their competency in practising KMC in fear of fathers suffocating the LBWIs. However, male involvement was a challenge at the health facility because of lack of privacy and males were denied access to KMC room, this prevented spouses supporting KMC.^43^ Kangaroo mother care practice was viewed as a hinderance to social obligations or responsibilities and when mothers abided by social and gender responsibilities KMC practice became a challenge.^44,45,55^ Most of the mothers relied on their husbands to make decisions. Lack of women empowerment and their low decision-making power hindered KMC acceptance and utilisation.^36,46,54^

#### Time and timing

Skilled health workers supported the early initiation of KMC, this facilitated KMC utilisation.^45,49^ The time taken to stabilise the LBWI, cultural or traditional belief of waiting for the umbilical cord to fall off and perception that KMC consumes time for house chores, contributed to late KMC utilisation.^38,43,46,47,49^ Limited family visiting hours and long hospital stay prevented family support and encouragement, these barriers facilitated absconding.^43,46^ In Africa KMC was regarded as a luxury practice and a waste of time as it was perceived as the practice for the whites and time spent in KMC practice could be used to source money or food for the family.^36^ Lack of KMC health education at the antenatal clinic and on-going KMC health education by the health workers during the hospital stay hindered mothers to practice KMC.^39,43,46,53,54^

#### Traditional and cultural norms

The open blouse of newly delivered mothers suits the dressing during KMC practice, however, in some cultures in India KMC dressing dishonoured cultural and traditional dressing. The cultural norm of mother–infant confinement days after delivery enhanced mother’s concentration on the infant and the cultural belief to keep the infants warm and less bathing promoted KMC utilisation.^36,47^ However, socially and culturally KMC was considered a new practice, hence, unwillingness to use new practices hindered KMC utilisation.^37,40^ In some cultures in Bangladesh, Ethiopia, India and Ghana, KMC practice was culturally not accepted as traditionally carrying the infant at the front was considered a taboo.^37,38^ Some mothers felt that prolonged infant holding brought a dependency habit in an infant that might disturb mothers from doing daily house chores and above all else ‘malnourished’ baby (LBWI) had to be fed and not kept in KMC position.^36,47^ Mothers who gave birth to LBWIs felt less of women and feeling of incompetence by giving birth to a LBWI, brought the fear of being ridiculed by the society, hence it prevented KMC utilisation.^36,37,43,49^ Culturally relatives influenced decision making in consenting to practice KMC, which barred KMC utilisation in time or not at all.^46^

## Discussion

Discussion of this study will be based on the summarised factors that influence and/or hinder KMC practice on a personal, facility and community operational and social levels. These factors were categorised as individual, systems Systems-facility and community levelsand social levels, respectively, see Table 5.

**TABLE 5:** Summary of the facilitating factors and barriers to kangaroo mother care utilisation by parent of low birthweight infants in low- and middle-income countries, 1990–2020.

Theme	Individual level		Systems level		Social level	
	Facilitators	Barriers	Facilitators	Barriers	Facilitators	Barriers
Access	KMC preference Affection towards the LBWIs Mother–infant bonding Maternal confidence/will to practice KMC Availability of skilled KMC health workers	The season of the year Privacy, Home delivery Low self-esteem and lack of confidence Felt less of women for having LBWIs	Availability of KMC providers, resources, Nurses’ empathy Hospital delivery: prompt KMC uptake	Home delivery: late/delayed KMC initiation Privacy: non-inclusion of males in KMC unit set up	-	Cultural association of infants skin rash to mother–infant skin contact
Buy-in	KMC knowledge Perceived and experienced KMC benefits Health seeking behaviour	Lack of knowledge on KMC protocol and safety by the PLBWIs, family members and community influential members Maternal, attitude towards KMC	KMC awareness	Non KMC awareness Lack of male involvement Lack of privacy and the males not allowed in the KMC room	-	-
Coordination and collaboration	-	-	Follow-up at the facility-based KMC KMC awareness through community sensitisation Educating the key influential community members Incorporating mothers in decision making on LBWIs’ care Good nurse–mother relationship	Non follow-up at community-based KMC	-	-
Medical issues	-	KMC perceived not safe and causes infection and neck deformity	-	Medical stabilisation of LBWI perceived as restriction to KMC initiation	-	-
Motivation	Mother–infant bonding Perceived, observed and experienced KMC outcomes	Experienced and perceived discomforts to the parent and/or LBWI associated with KMC	Use of KMC expert clients Return demonstration, Displayed KMC pictures/dolls KMC pictorial presentations and photographs Recreation activities Managing postpartum pains	-	PLBWI ridiculed by the family and community	-
Social support, gender obligation and empowerment	-	-	KMC support and encouragement Male involvement Woman empowerment: decision-making Nurses’ willingness to educate PLBWIs	-	Family and community support with KMC practice Prior identified support system KMC support groups facilitated KMC utilisation Women empowerment	Lack of family support Lack of women empowerment
Time and timing	-	-	Early KMC initiation, Ongoing KMC education at facility-based care	Lack of KMC health education at community based KMC Limited family visiting hours Long hospitalisation stay	-	KMC consumes time for house chores KMC waste of time: KMC is for the whites
Traditional/cultural norms	-	-	-	Type of wrap: customised	Mother–infant confinement Type of wrap: traditional chitenje	KMC hinders social obligations Cultural/traditional belief of waiting for the umbilical cord to fall off before KMC started KMC considered as taboo

PLBWIs, parent of low birthweight infant; KMC, kangaroo mother care; LMICs, low- and middle-income countries; LBWI, low birthweight infants.

### Individual level

Maternal love, confidence coupled with health-seeking behaviour prompted PLBWIs to conduct KMC regardless of the circumstances,^36,38,40,44,46,49,55^ which brought an individual sense of mother–infant bonding and promoted KMC practice.^40,43^ Associating KMC with foreign practice, cosmetic purposes, lack of interest with KMC and a sense of low self-esteem affected the zeal to practice KMC.^36,37,38,40^ Previous reviews concur that maternal natural instinct to protect their infants promote KMC utilisation, which enhance self-esteem, mother–infant bonding and affection towards the LBWIs.^55,57^ Therefore, individual convictions on KMC practice affected the utilisation of KMC, as such health-seeking behaviour and social behaviour changes need to play a role in KMC practice regardless of PLBWLs opinions on LBWIs and KMC service.

It is important to target KMC beneficiaries (pregnant mothers, post-natal mothers and women of the childbearing age) with KMC information. This may influence their health seeking behaviour and help them make informed choices towards KMC utilisation. Precious reviews indicate that targeted education influences health seeking behaviour and attitudes towards an intervention.^57,58,59^ Therefore, individualised KMC health education may influence KMC utilisation by PLWBIs without necessarily waiting to verify the workability of KMC by observing or experiencing its benefits, as this study indicated.

### Systems – facility and community levels

The constant availability of skilled KMC health workers in KMC units and nurses’ willingness to educate PLBWIs about KMC facilitated acquisition of KMC knowledge by PLBWIs on KMC protocol and safety. This enhanced the parental choice on KMC utilisation options and facilitates KMC preference and early initiation of KMC.^36,37,38,40,45,46,47,49,50^ Other studies concur with this study’s findings that availability of KMC providers and nurses’ attitude towards KMC play a role in KMC knowledge dissemination and hasten KMC initiation by parents.^25,57^ Mothers who delivered at the hospital had access to KMC providers, KMC knowledge and protocols, safety and medical stabilisation of the LBWIs before KMC initiation, which facilitated early KMC initiation and promoted infants’ survival, which was not the case with home delivery.^36,40,55^ Studies show that hospital delivery not only provides the mother with safe delivery services but also access to maternal and neonatal interventions that enhance their survival as LBWIs are stabilised before KMC initiation, which is not the case with home deliveries.^25,55^ Although, stabilising medically challenged infants was viewed as the contributing factor to late initiation of KMC.^38,43,46,49,55^ Lack of knowledge on KMC protocol and safety by the PLBWIs, family members and community influential members hindered KMC utilisation.^37,38,47,50^ Two reviews concur with our finding that lack of knowledge by the family members contributes to stigma towards LBWIs and non-support of KMC.^25,60^ Furthermore, lack of KMC health education and ongoing education at facility and community antenatal clinics infringed pregnant women and PLBWIs from acquiring KMC knowledge prenatally and postnatally, respectively.^39,43,45,46,53,55^ A review narrates that investing in KMC tailored health education will enhance KMC knowledge and utilisation.^25^ Kangaroo mother care awareness through community sensitisation and educating the key influential community members influenced KMC acceptability, accessibility utilisation and support.^37,40,46,49,50^ Reviews concur that community awareness on KMC improves its utilisation by the parents and the community.^60,61^

This study indicated that anticipated, perceived, observed and experienced KMC outcomes,^36,38,40,43,44,45,46,47,48,49^ brought satisfaction, comfortability and contentment in PLBWIs, which promoted KMC utilisation.^40,43,45,46^ However, the experienced and perceived discomforts to the parent and/or LBWI associated with KMC practice dented KMC as an unsafe practice, to LBWIs.^36,37,43,47,48,49^ This finding is similar to other review findings that positive perception on KMC benefits promote KMC utilisation and lack of knowledge on KMC, a sense of discomfort and a feeling of LBWI being hurt in KMC position brought negative perceptions on KMC.^25,57,60^ Male involvement marked the backbone of family support, unfortunately, a lack of privacy and the males not allowed in the KMC room are the drawbacks to males being involved in the care of their LBWIs,^40,43^ hence barring fathers from supporting and utilising KMC. Reviews have indicated that engaging or teaching fathers in KMC promotes KMC support whilst lack of male inclusion and involvement in KMC interventions and lack of privacy in the hospital setting prevents fathers and grandfathers from supporting KMC, which is one of the reasons for discontinuing KMC utilisation.^23,57^ Limited family visiting hours, lack of family support in KMC practice barred family members from relieving mothers from the discomforts accompanying KMC practice, hence the increase of KMC abscond associated with fatigue, boredom and long stay in hospital.^36,37,46,47^ Reviews concur with short visiting hours as a barrier to KMC support by family members and an extension of the visiting hours or frequent family visits promote KMC support, hence it enhances utilisation.^25,60^

Implementation of coordinated, collaborative interventions and follow-up at the facility and community-based KMC and nurses’ guidance on KMC practice at home improved the uptake of KMC. In addition, updating PLBWIs on their infants’ condition, incorporating mothers in decision making on LBWIs’ care and good nurse–mother relationship, motivated PLBWIs to utilise KMC.^43,50^ Reviews concur with this study’s finding in the sense that engaging parents in KMC promotes nurse–parent relations and motivates mothers to resume responsibilities in utilising KMC.^57^

This study indicated that motivational interventions, use of KMC expert clients, return demonstration, KMC pictures or dolls, KMC pictorial presentations and photographs, recreation activities and managing postpartum pains, influenced PLBWIs to utilise KMC.^36,37,46,47^ This finding concurs with other review in the sense that KMC practical demonstration promotes its utilisation.^57^

### Social level (household and community level)

Parent of low birthweight infants were ridiculed for giving birth to LBWIs, which brought fear unto the mothers and they felt less of women for having LBWIs that hindered KMC utilisation.^36,37,43,49^ Previous studies concur with the finding that stigma towards PLBWI brought anxiety and sense of guilt that lead to not wanting to keep the baby, thus hindering KMC utilisation.^25^ Furthermore, the cultural association of infants skin rash to mother–infant skin contact prevented KMC optimal utilisation.^36,37,47^ Family and community support with KMC practice, household chores and encouragement influenced KMC utilisation.^36,43,44,45,46,47,48,49,50^ Reviews agree to our finding that supportive environment promotes PLBWIs to utilise KMC, whilst family negative remarks or stigma to PLBWIs deters KMC utilisation.^25,57^ Furthermore, prior identified support system and KMC support groups facilitated KMC utilisation.^36,45^

Kangaroo mother care practice is viewed as a hindrance to social obligations or responsibilities and when mothers abided by social and gender responsibilities KMC practice became a challenge.^44,45^ Kangaroo mother care is identified as an intervention that consumes time for house chores as the mother had limited time to do house chores and the cultural or traditional belief of waiting for the umbilical cord to fall off before KMC started delayed initiation of KMC.^47^ The reviews agree that KMC lobbies mother’s time to attend to family responsibilities and that KMC promotes infants dependency to KMC position, which in turn KMC is not utilised consistently.^57^ In some cultures, the KMC protocols correlated with their cultural norms and beliefs, which promoted KMC utilisation.^36,47^ However, in some cultures KMC is still considered a taboo and adopting it remains a challenge.^36,37,38^ Reviews show that cultural factors affected parents from accepting KMC utilisation, hence deterred KMC utilisation.^57,60^ Furthermore, cultural influence on women empowerment in decision-making influenced KMC utilisation.^36,46^ In some cultures, KMC practise was viewed as care that needed privacy and counteracted with women’s social responsibilities and obligations, which disturbed family routines and increased household workload, hence preventing KMC utilisation.^43,47^ Another review agrees that fathers prefer to support KMC utilisation at home as compared with facility based KMC because of discomfort from spectators in the hospital.^57^

### Strengths and limitations

This scoping review was vigorously conducted to comprehensive identify facilitating factors and barriers to KMC utilisation by PLBWIs in LMICs. The studies were searched in SciELO, Google Scholar, JSTOR, LILACS, Academic search complete, CINAHL with full text, Education source, Health Source: Nursing/Academic Edition, Medline with full text, Medline and PubMed databases. This review demonstrated a significant gap in the literature on facilitating factors and barriers to KMC utilisation by PLBWIs in LMICs. Using the Medical Subject Heading terms and the designed charting form allowed the identification and inclusion of relevant studies, which formed the strength of this study. Although rigorous steps were followed throughout this review, we may have omitted relevant studies that might not have been accessible through the databases searched, not published at all and not published in languages other than English. Therefore, further reviews could focus on inclusion of studies published irrespective of the English language, date of publication and design and/or methodology. Although most of the studies focused on the health professionals’ perspectives on barriers and facilitating factors to KMC utilisation in LMICs, this study focused on PLBWI’s views on the same. Therefore, we recommend similar studies in the future to explore the facilitating factors and barriers to KMC utilisation by the other cadres.

## Conclusion and recommendations

This review recommends the adoption of educative and care strategies on KMC accessibility and utilisation targeting mothers and the community may promote KMC uptake. Prior knowledge on the availability of KMC services is crucial to KMC practice as it enhances informed decision-making and utilisation of KMC. Cultural and traditional beliefs need to be factored in the KMC utilisation strategies as they play a role in KMC acceptability and utilisation. Kangaroo mother care follow-up and support rendered to PLBWIs enhance KMC confidence, comfortability, acceptability and utilisation. Adequate KMC support may display a positive lived experience that may promote KMC practice by PLBWIs and to mothers faced with the same situation KMC awareness, demonstrations and return demonstration on KMC and involvement of community key players may enhance social and traditional acceptance of KMC practice. Kangaroo mother care awareness should be disseminated by trained and skilled health personnel to enhance hospital delivery and community or mothers’ confidence in KMC service.

In addition, as implication to the study’s findings, we recommend the development of these suggested interventions, initiate KMC open days to showcase KMC models that will share their KMC lived experiences and help clear misconceptions related to LBWI and KMC. Evidentially, women empowerment can enhance prompt decisions regarding KMC access and utilisation. Thus, tabling strategies that strengthen women empowerment, that is, addressing gender-based issues, may improve KMC access and utilisation by PLBWIs.

Government, implementing partners and trained community leaders should advocate for either redefining, modifying or dropping harmful cultural or traditional tendencies on LBWIs. Our study identified that LBWIs and KMC practices are regarded as a taboo, hence PLBWIs were barred from utilising KMC. The KMC unit should be redesigned in a manner that is male sensitive, to accommodate and promote male involvement, family centered care and spouse support with KMC utilisation.

In conclusion, this study revealed that PLBWIs play a crucial role in the success of KMC service. As such, on-going prenatal, antenatal and post-natal KMC health education, community sensitisation and awareness, engaging, collaborating and coordinating community key structures in KMC may improve KMC utilisation by the PLBWIs. Therefore, it is important for the policy or decision-makers, implementers, funders, KMC guidelines developers to focus on inclusion of KMC uptake strategies by the PLBWIs, which is crucial in the reduction of morbidity and mortality related to LBWIs’ complications. The outcomes of this scoping review may inform future research and further identify the evidence-based interventions, which may inform policies and guidelines, to improve KMC utilisation in LMICs, prevent LBWI deaths and contribute towards the SDG 3 goal of 12 neonatal deaths per 1000 live births per country by 2030.^3,8^

## Figures and Tables

**FIGURE 1-A1:**
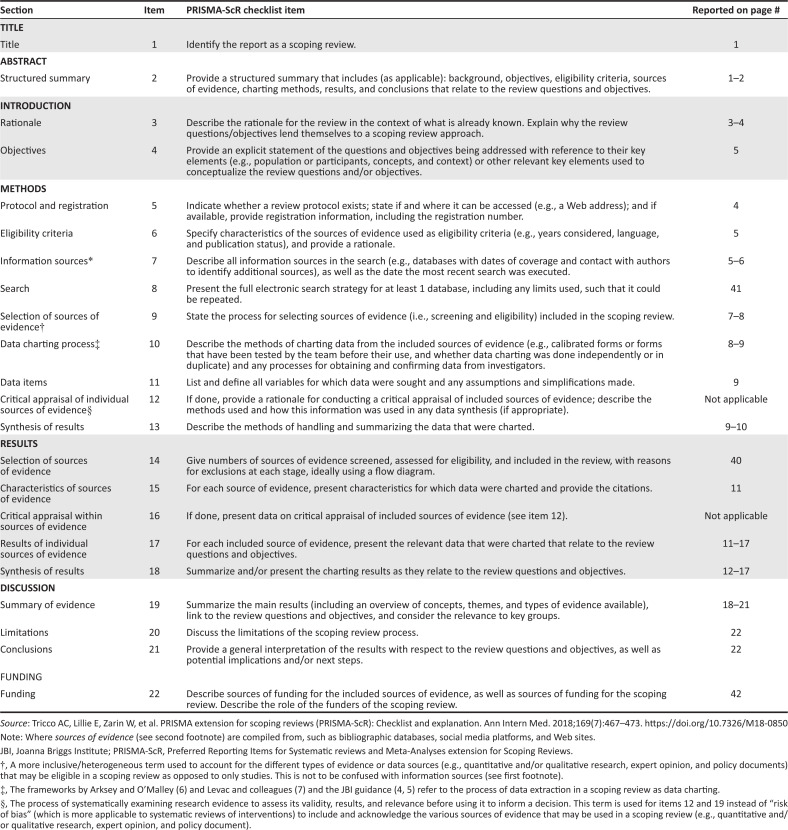
Preferred Reporting Items for Systematic reviews and Meta-Analyses extension for Scoping Reviews (PRISMA-ScR) Checklist.
